# Mechanisms of Nifedipine-Downregulated CD40L/sCD40L Signaling in Collagen Stimulated Human Platelets

**DOI:** 10.1371/journal.pone.0127054

**Published:** 2015-05-13

**Authors:** Tso-Hsiao Chen, Ching-Yu Shih, Wen-Lin Hsu, Tz-Chong Chou

**Affiliations:** 1 Division of Nephrology, Department of Internal Medicine, Wan Fang Hospital, Taipei Medical University, Taipei, Taiwan; 2 Graduate Institute of Life Sciences, National Defense Medical Center, Taipei, Taiwan; 3 School of Medicine, Tzu Chi University; Department of Radiation Oncology, Buddhist Tzu Chi General Hospital, Hualien, Taiwan; 4 Institute of Medical Sciences, Tzu Chi University, Hualien, Taiwan; 5 Department ofBiotechnology, Asia University, Taichung, Taiwan; 6 China Medical University Hospital, China Medical University, Taichung, Taiwan; Boston University, UNITED STATES

## Abstract

The platelet-derived soluble CD40L (sCD40L) release plays a critical role in the development of atherosclerosis. Nifedipine, a dihydropyridine-based L-type calcium channel blocker (CCB), has been reported to have an anti-atherosclerotic effect beyond its blood pressure-lowering effect, but the molecular mechanisms remain unclear. The present study was designed to investigate whether nifedipine affects sCD40L release from collagen-stimulated human platelets and to determine the potential role of peroxisome proliferator-activated receptor-β/-γ (PPAR-β/-γ). We found that treatment with nifedipine significantly inhibited the platelet surface CD40L expression and sCD40L release in response to collagen, while the inhibition was markedly reversed by blocking PPAR-β/-γ activity with specific antagonist such as GSK0660 and GW9662. Meanwhile, nifedipine also enhanced nitric oxide (NO) and cyclic GMP formation in a PPAR-β/-γ-dependent manner. When the NO/cyclic GMP pathway was suppressed, nifedipine-mediated inhibition of sCD40L release was abolished significantly. Collagen-induced phosphorylation of p38MAPK, ERK1/2 and HSP27, matrix metalloproteinase-2 (MMP-2) expression/activity and reactive oxygen species (ROS) formation were significantly inhibited by nifedipine, whereas these alterations were all attenuated by co-treatment with PPAR-β/-γ antagonists. Collectively, these results demonstrate that PPAR-β/-γ-dependent pathways contribute to nifedipine-mediated downregulation of CD40L/sCD40L signaling in activated platelets through regulation of NO/ p38MAPK/ERK1/2/HSP27/MMP-2 signalings and provide a novel mechanism regarding the anti-atherosclerotic effect of nifedipine.

## Introduction

The mortality of cardiovascular diseases including atherosclerosis characterized by vascular inflammation and endothelial damage/dysfunction remains the leading cause of death all over the world [1,2]. Nifedipine, a dihydropyridine-based L-type calcium channel blocker (CCB), is widely used for hypertension therapy. Moreover, nifedipine has been reported to prevent the progression of atherosclerosis [3], but the underlying molecular mechanisms remain unclear. The atheroprotective effect of nifedipine is proposed to be independent of calcium channel blocking activity [3], suggesting that other actions of nifedipine may be involved.

The interaction between CD40 and its ligand (CD40L) results in promoting vascular disorders and atherothrombosis by activating inflammatory and coagulant responses [4, 5]. The CD40L (CD154) and CD40 are members of tumor necrosis factor (TNF) and TNF-receptor family, respectively. Increased plasma soluble CD40L (sCD40L) level was observed in patients with acute coronary syndrome [6]. Blocking CD40L actions with specific antibody greatly attenuated the atherosclerotic lesions in hyperlipidemic mice [7]. Moreover, CD40L also stimulates platelet activation and stabilizes arterial thrombi through glycoprotein IIb/IIIa ligand-dependent mechanism [8,9]. These results support the important role of CD40L in the pathogenesis of atherosclerosis. Currently, platelets have been believed to be important cells in modulating inflammatory responses by releasing several pro-inflammatory and pro-atherogenic components such as CD40L. In unstimulated platelets, CD40L is stored in α-granules; upon activation, CD40L is rapidly released from α-granules and translocated to the surface membrane of platelets. Then, the membrane-bound CD40L is cleaved from the membrane by matrix metalloproteinases (MMPs), and released as soluble CD40L (sCD40L) [10]. Notably, platelets are the main source of sCD40L, and at least 95% of circulating sCD40L comes from platelets [11]. Therefore, targeting surface CD40L expression and sCD40L release can be a promising strategy for alleviating atherosclerosis by blocking the linkage between platelet activation, inflammation and thrombosis.

Peroxisome proliferator-activated receptors (PPARs) belonging to ligand-activated transcription factors modulate many metabolic processes, including lipid metabolism, and glucose homeostasis [12]. In addition, other effects of PPARs such as anti-atherogenic, anti-inflammatory and antiplatelet activities have been reported [13–15]. Although, platelets are anucleated cells derived from megakaryocytes, they also contain transcription factors such as PPARs. There are three PPAR isoforms (PPAR-α, PPAR-β/γ, and PPAR-γ) existed in human platelets. Clinical and *in vitro* studies have indicated that treatment with PPAR-γ agonists significantly decreased the serum levels of sCD40L in patients with coronary artery disease and sCD40L release from thrombin-stimulated platelets [16,17]. Interestingly, the serum sCD40L levels in the hypertensive patients with type 2 diabetes were reduced significantly after 3 months of nifedipine treatment [18], suggesting that the vascular protective effect of nifedipine may be associated with suppression of sCD40L release. However, whether nifedipine affects the CD40L/sCD40L cascade in human platelets remains unknown. Based on recent study showing that nifedipine is a PPAR-β/-γ agonist in human platelets [19], we hypothesize that the regulatory effects of nifedipine on CD40L/sCD40L signallings in human platelets are mediated by PPAR-β/-γ -dependent pathway.

## Materials and Methods

### Reagents

Collagen (type I, equine tendon) was obtained from Chrono-Log Corporation. (Broomall, PA, USA). RIPA buffer was obtained from Pierce Biotechnology Inc (Meridian Rd, Rockford, USA). The enzyme-linked immunosorbent assay (ELISA) kit of cyclic GMP, and PPAR-γ antibody were purchased from Cayman Chemical Company (Ann Arbor, MI, USA). The GSK0660 and GW9662 were purchased from Tocris (Avonmouth, Bristol, UK). ECL reagent was purchased from Upstate Biotechnology (Lake Placid, NY, USA). The antibodies of PPAR-β, and β-actin were purchased from Senta cruz biotechnology (Santa Cruz, CA, Europe). The phospho-HSP27, total-HSP27, phospho-p38 mitogen-activated protein kinase (p38MAPK), total-p38MAPK, phospho-ERK1/2, total-ERK1/2 antibodies were purchased from Cell Signaling Technology (Beverly, MA, USA). Nifedipine was purchased from Sigma Chemical Company (St. Louis, MO, USA) and dissolved in dimethylsulfoxide (DMSO) followed by dilution with Tyrode solution, and the final concentration of DMSO was fixed at 0.1%. Other chemical reagents were purchased from Sigma Chemical Company.

### Platelet aggregation

Venous blood drawn from healthy volunteers was mixed with ACD solution (75 mM tri-sodium citrate, 42 mM citric acid and 136 mM glucose, pH 5.2) and centrifuged at 160g, 25°C for 10 min to obtain platelet rich plasma (PRP). Then, the PRP was suspended in Tyrode's solution (pH 7.4) to prepare washed platelets and adjusted the platelet concentration to the 3.0x10^8^ platelets/ml as previously described [19]. The study and the written informed consent were approved by the Ethical Committee of National Defense Medical Center in accordance with the declaration of Helsinki for experiments involving humans. All subjects sign informed consent for their participation in this study.

### Determination of platelet surface CD40L expression and sCD40L release

Washed platelets were preincubated with 2.5 μl FITC labeled monoclonal anti-CD40L antibody (Abcam, Cambridge, Ma, USA) and various drugs or solvent control for 6 min at 37°C, followed by addition of collagen (10 μg/ml) for 10 min. The reaction was stopped and fixed by addition of 1% paraformaldehyde 500μl for 5 min at 37°C. The sample was centrifuged at 10,000g for 3 min and suspending the pellet with 1ml PBS buffer. Then, the fluorescence intensity of 20,000 platelets per sample was analyzed using a flow cytometer (BD Biosciences, San Jose, CA, USA). For determination of sCD40L release, washed platelets were treated with various drugs followed by centrifugation at 10,000g for 5 min to obtain supernatants that were used to measure the amounts of sCD40L by ELISA Kit (PeproTech, Rocky Hill, New Jersey, USA)

### Determination of cyclic GMP formation

Washed platelets were preincubated with various drugs or solvent control followed by addition of collagen (10 μg/ml) for 6 min at 37°C and stopped the reaction by adding EDTA (10 mM) and immediately boiling for 5 min. After centrifugation at 10,000g for 5 min at 4°C, the cyclic GMP content of supernatants was measured by an ELISA kit.

### Determination of nitrate+nitrite (NOx) formation

Washed platelets were preincubated with various drugs or solvent control for 3 min at 37°C, followed by addition of collagen (10 μg/ml) for 6 min and centrifugation at 10,000g for 5 min at 4°C. The amount of NOx in the supernatants was measured by a Sievers Nitric Oxide Analyzer (Sievers 280 NOA, Boulder, CO, USA). Nitrate concentrations were calculated by comparison with standard solution of sodium nitrate.

### Western blotting

Platelets (3×10^8^/mL) of various groups were incubated with RIPA buffer (150 mM NaCl, 0.5% sodium deoxycholate, 0.1% sodium dodecyl sulfate, 50 mM Tris, pH 8.0) containing a mixture of protease and phosphatase inhibitors (Sigma Chemical Co, St. Louis, MO, USA). The cell lysates were heated at 95°C for 10 min and proteins (20 μg) were separated in 8% sodium dodecyl sulfate (SDS)-polyacrylamide gel and electrotransferred by semi-dry transfer (Bio-Rad Laboratories, Inc., Hercules, CA, USA). Then, various primary antibodies were incubated with transferred membranes for 1.5 h followed by addition of peroxidase-conjugated secondary antibody in PBS-Tween 20 for 1 hrs. The immunoreative bands of target genes were detected with an enhanced chemiluminescence (ECL) kit (Amersham International Plc., Buckinghamshire, UK) with reference to a cytoplasmic protein (β-actin).

### Measurement of reactive oxygen species (ROS) production

For determination of H_2_O_2_ formation, washed platelets were preincubated with 10 μM 2’,7’- dichloro-dihydro-fluorescein diacetate (DCFH-DA) for 30 min at 37°C in dark. After extensive washing with PBS, 240 μl of platelets **(**2x10^8^/ ml) were added to 96-well plates containing 5 μl of various concentrations of drugs or solvent control and incubation for 3 min followed by addition of collagen (10 μg/ml) for another 10 min and the emitted DCF fluorescence was photographed with a fluorescence microscope (Leica, Welzar, Germany) as previously described [20]. For measurement of O_2_^-^ production, platelets were treated with nifedipine or solvent control at 37°C for 3 min followed by addition of nitroblue tetrazolium (NBT) (2 mg/ml) and collagen for 1.5 h. Then, the platelet suspension was centrifuged at 10,000 g for 5 min to collect the pellets and resolved in 5% Triton-X 100. The absorbance at 560 nm was detected with a spectrometer.

### Zymography

MMP-2 activity was determined by using gelatin zymography. After treatment with various drugs, the platelet supernatant was collected and electrophoresed on an 8% polyacrylamide gel containing 0.1% gelatin. Then, the gel was washed for 30 min 2 times in washing buffer (2.5% Triton X-100) and incubated in buffer (1% NaN3; 2M Tris- HCl, pH 8.0; 1M CaCl_2_) at 37°C for 16 h with shaking and subsequently stained with Coomassie brilliant blue R-250. The presence of MMP-2 gelatinolytic activity was identified as clear zones of lysis against a blue background after destaining. The proteolytic activities were quantified from gelatin gels by densitometric scanning using a Bio-Rad GS-690 Imaging Densitometer.

### Statistical analysis

The experimental results were expressed as means and their standard errors. Statistical analysis was performed with Student’s t test or one-way ANOVA with a Dunnett’s post hoc test. A value of *P* < 0.05 indicated a significant difference.

## Results

### Nifedipine inhibited the surface CD40L expression and sCD40L release

**Fig 1:**
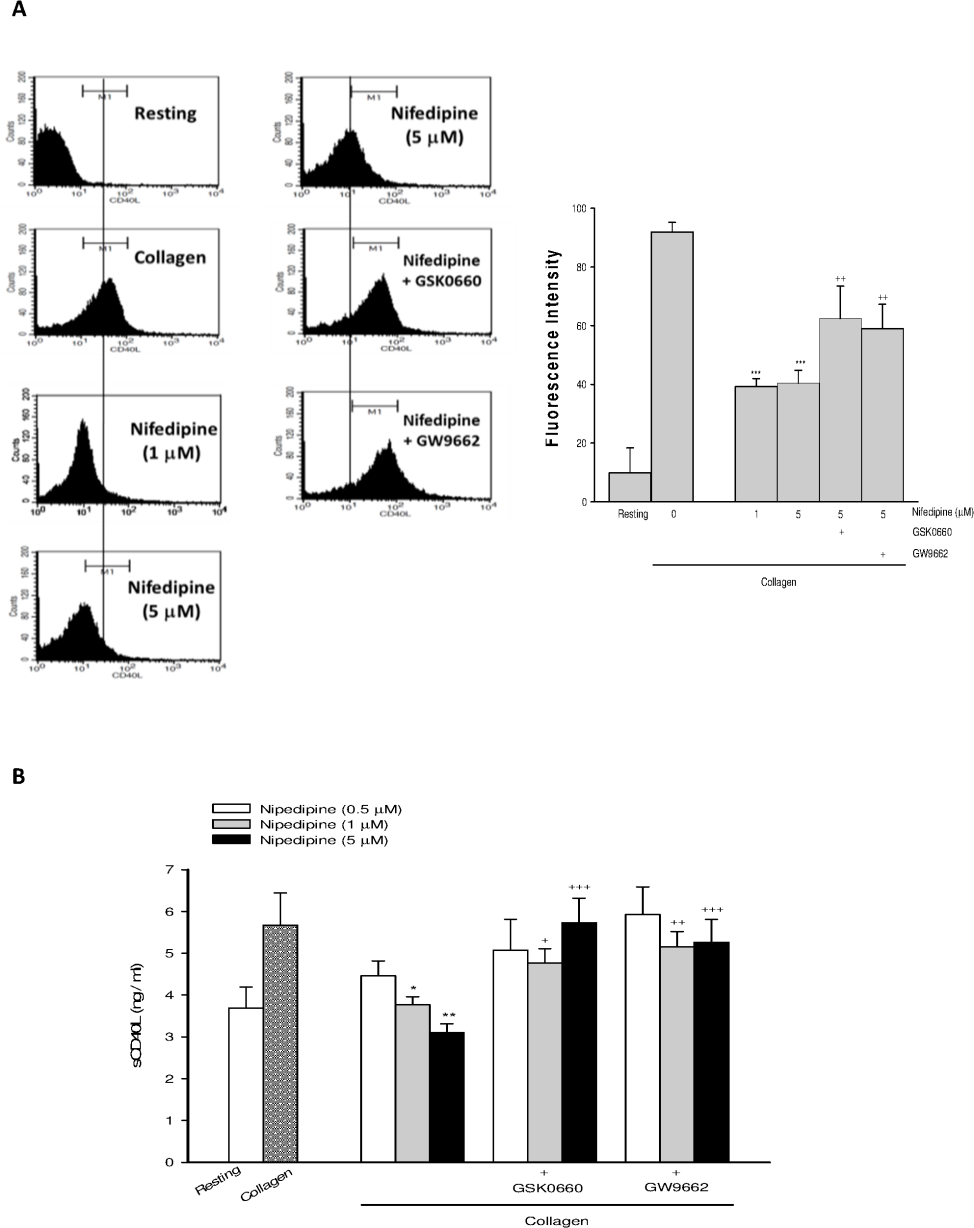
Effects of nifedipine on the membrane CD40L expression and sCD40L release. Washed platelets were preincubated with various doses of nifedipine or nifedipine combined with GSK0660 (5 μ M) or GW9662 (5 μ M) for 3 min, followed by addition of collagen (10 μ g/ml) for 6 min. The platelet surface CD40L expression (A) and the levels of sCD40L (B) were determined. Data were presented as means ± SEM (*n* = 5). ^*^ *p* < 0.05, ^**^ *p* < 0.01, ^***^ *p* < 0.001 compared with the collagen alone group; ^+^ *p* < 0.05, ^++^ *p* < 0.01, ^+++^ *p* < 0.001 compared with respective collagen+nifedipine-treated group.

Nifedipine dose-dependently inhibited collagen-induced elevation of platelet surface CD40L expression (Fig 1A) and sCD40L release (Fig 1B), whereas the inhibition was greatly reversed by co-treatment with GSK0660 (5 μM), a PPAR-β antagonist, or GW9662 (5 μM), a PPAR-γ antagonist, confirming the role of PPAR-β/-γ.

### PPAR-β/-γ involved nifedipine-mediated NO and cyclic GMP formation

**Fig 2:**
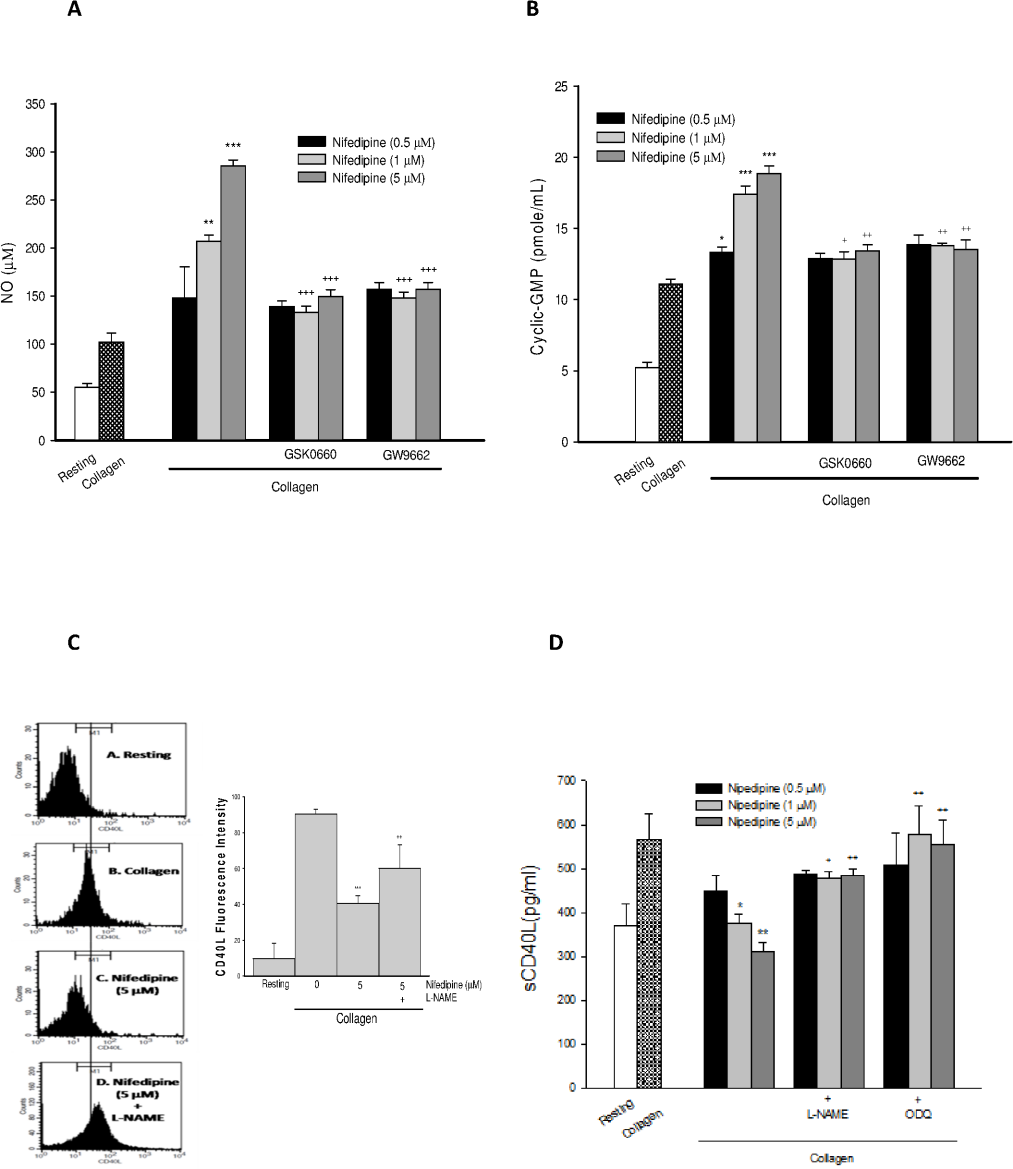
Effects of PPAR-β/-γ antagonists on nifedipine-mediated NO/cyclic-GMP formation and CD40L/sCD40L cascade. Platelets were incubated with various drugs, followed by addition of collagen. The levels of NOx (A) cyclic GMP formation (B) platelet surface CD40L expression (C) and sCD40L concentrations (D) were determined. Date were expressed as means ± SEM (*n* = 5). ^**^ *p* < 0.01, ^***^ *p* < 0.001 compared with collagen alone group; ^+^ *p* < 0.05, ^++^ *p* < 0.01, ^+++^ *p* < 0.001 compared with respective collagen+nifedipine-treated group.

Nifedipine dose-dependently increased NO (Fig 2A) and cyclic GMP formation (Fig 2B) in collagen-stimulated platelets, whereas the effects were strongly diminished by PPAR-β/-γ antagonists, suggesting the involvement of PPAR-β/-γ in NO and cyclic GMP formation. Meanwhile, in the presence of NG-nitro L-arginine methyl ester (L-NAME, 100 μM), an inhibitor of NOS or 1H-[1,2,4] oxadiazolo[4,3-a] quinoxalin-1-one (ODQ, 10 μM), an inhibitor of soluble guanylyl cyclase (sGC), dimished the decrease of CD40L expression and sCD40L release by nifedipine, significantly (Fig 2C and 2D).

### PPAR-β/-γ regulated nifedipine-mediated p38MAPK/ERK1/2/HSP27 phosphorylation

**Fig 3:**
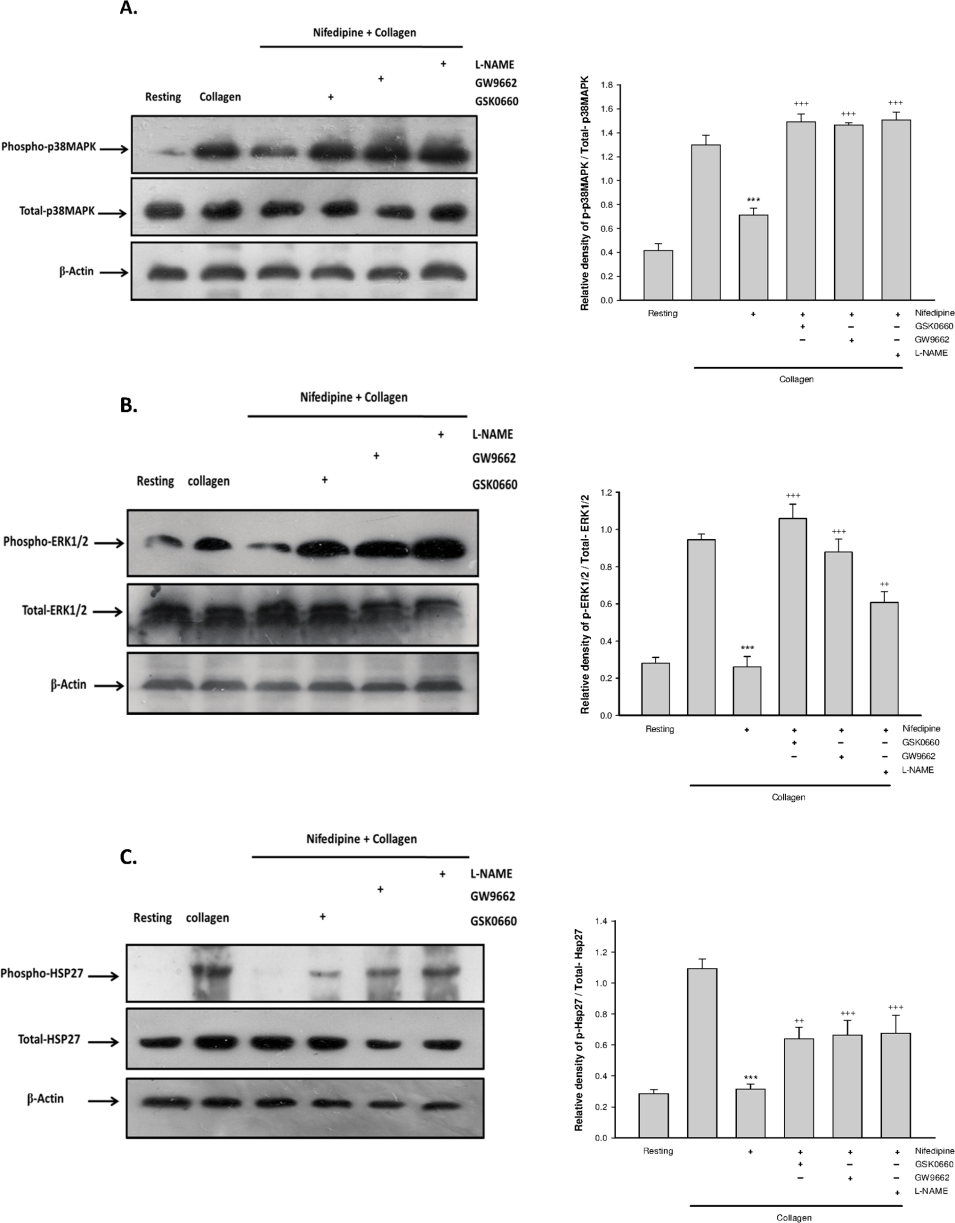
Effects of PPAR-β/-γ antagonists on nifedipine-mediated phosphorylation of P38MAPK, ERK1/2 and HSP27. Platelets were pretreated with 5 μ M nifedipine or nifedipine combined with various drugs for 3 min followed by addition of collagen for 6 min. The expression of phospho-P38MAPK and total-P38MAPK (A), phospho-ERK1/2 and total ERK1/2 (B), phospho-HSP27 and total-HSP27 (C) were determined. Date were expressed as means ± SEM (*n* = 5). ^***^ *p* < 0.001 compared with collagen alone group; ^++^ *p* < 0.01, ^+++^ *p* < 0.001 compared with respective collagen+nifedipine-treated group.

As shown in Fig 3, activation of platelets with collagen led to phosphorylation of p38MAPK, ERK1/2 and HSP27 and this alteration was markedly attenuated by treatment with nifedipine. However, the effects of nifedipine were effectively blunted when PPAR-.β/-γ antagonists or L-NAME was added simultaneously.

### PPAR-β/-γ contributed to nifedipine-mediated reduction of matrix metalloproteinase-2 (MMP-2) activity

**Fig 4:**
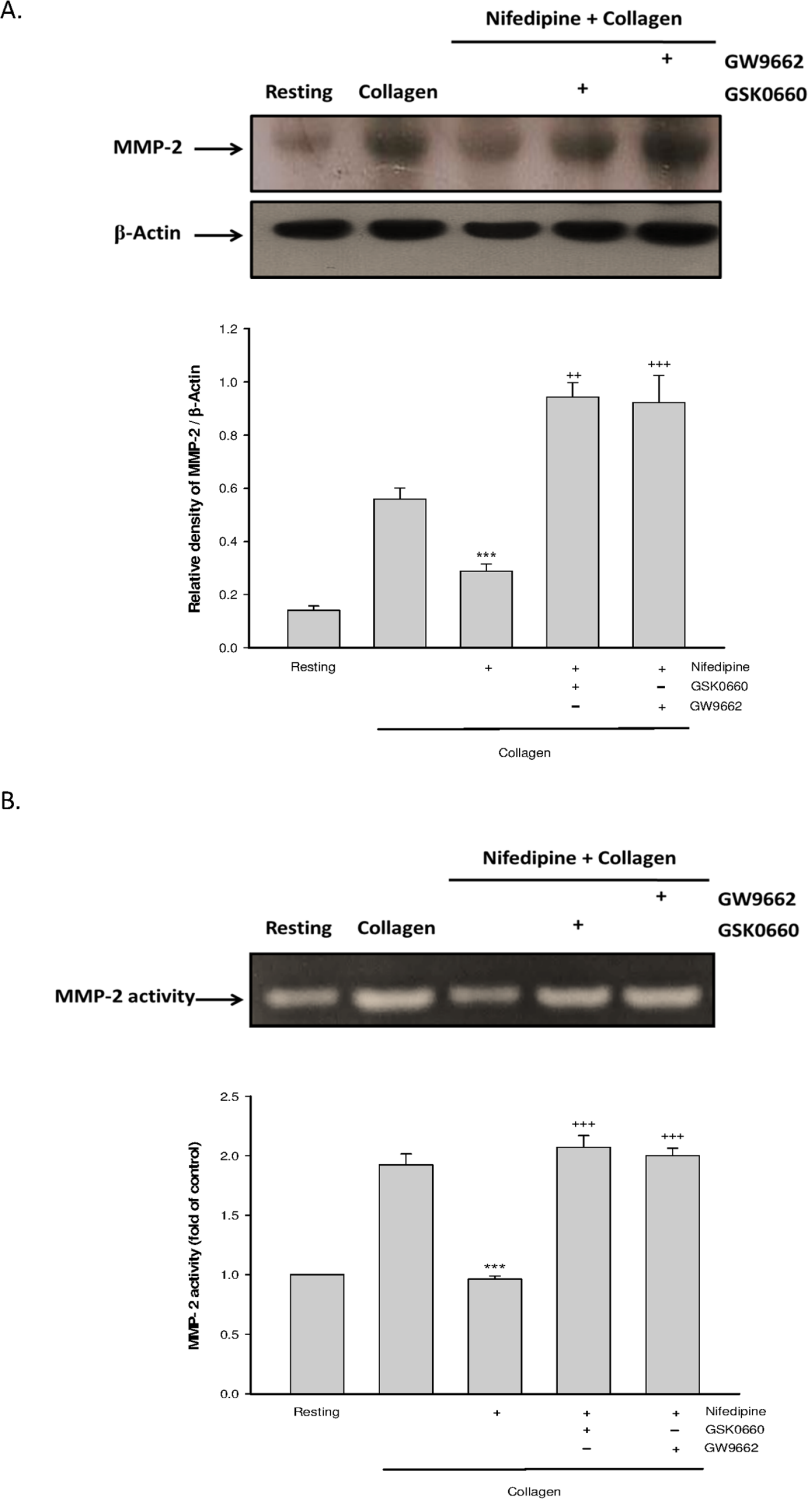
Effects of PPAR-β/-γ antagonists on nifedipine-mediated MMP2 activity. Platelets were treated with nifedipine (5 μ M), or nifedipine combined with various drugs for 3 min, followed by addition of collagen for 6 min. The MMP-2 expression (A) and MMP-2 activity (B) were determined. Date were expressed as means ± SEM (*n* = 5). ^***^ *p* < 0.001 compared with collagen alone group; ^++^ *p* < 0.01, ^+++^ *p* < 0.001 compared with respective collagen+nifedipine-treated group.

MMP-2 is a critical step for cleaving the membrane-bound CD40L leading to release of sCD40L [10]. Our data showed that collagen-induced rise of MMP-2 expression (Fig 4A) and activity (Fig 4B) were markedly inhibited by nifedipine, but the effects were completely diminished when platelets were co-treated with PPAR-β/-γ antagonists.

### PPAR-β/-γ involved nifedipine-mediated attenuation of ROS production

**Fig 5:**
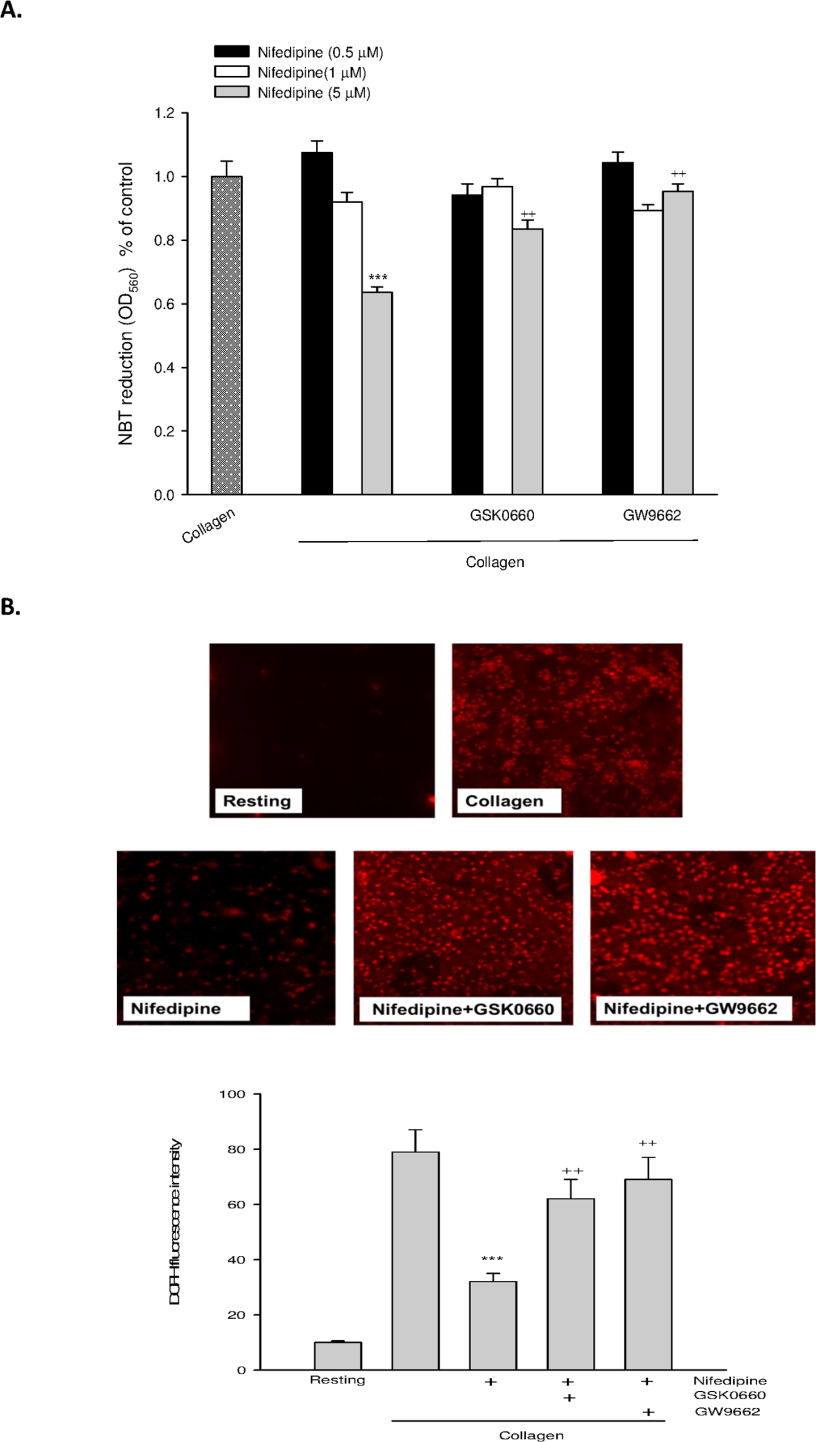
Effects of PPAR-β/-γ antagonists on nifedipine-mediated ROS production. Platelets were preincubated with nifedipine or nifedipine combined with GSK0660 or GW9662 for 3 min; then NBT or DCFH-DA was added, followed by collagen to measure superoxide (A) and hydrogen peroxide (B) formation respectively. Data were expressed as means ± SEM (*n* = 5). ^***^ *p* < 0.001 compared with collagen alone group; ^++^ *p* < 0.01 compared with respective collagen+nifedipine treated group.

Because ROS in particular hydrogen peroxide is a potent activator for MMP-2, we further determined the involvement of PPAR-β/-γ in nifedipine-mediated ROS formation. We found that treatment with nifedipine dose-dependently inhibited collagen-induced O_2_^-^ (Fig 5A) and H_2_O_2_ formation (Fig 5B) in platelets determined by NBT and DCFH-DA, respectively. Similarly, the antioxidative activity of nifedipine was greatly reduced by GSK0660 or GW9662.

## Discussion

The strong positive correlation between platelet hyper-reactivity accompanied by enhanced interaction with leukocytes and the development of atherosclerosis has been described [21]. Several inflammatory and prothrombotic mediators including CD40L stored in α-granules are rapidly translocated to the surface of platelets and released into the plasma when platelets are activated by agonists such as collagen or thrombin. Other small amount of sCD40L can be released from various cells including endothelial cells, and monocytes. The sCD40L has been regarded as a potent stimulator for promoting the attachment of leukocytes and subsequent inflammatory responses. CD40L also acts as a stimulator for platelet activation due to binding to platelet glycoprotein IIb/IIIa receptors [8]. Therefore, CD40L is a critical inducer for cardiovascular diseases by providing a link between the immune system, atherosclerosis and thrombosis [22], and the plasma level of CD40L has been considered an important marker of platelet activation and the prognosis in cardiovascular diseases. Since platelets are predominant source of circulating CD40L, the platelet-derived sCD40L release may be a potential target for alleviating vascular diseases. Although, the protective effect of nifedipine in vascular diseases is observed, the underlying molecular mechanisms remain to be elucidated. In the present study, we demonstrated that nifedipine inhibits sCD40L release and the surface CD40L expression in collagen-activated platelets, whereas the effects were markedly dampened by blocking PPAR-β/-γ activity. Moreover, the inhibitory effect of nifedipine on surface CD40L expression was also seen in thrombin-stimulated platelets (S1 Fig), indicating that the actions of nifedipine on CD40Ltranslocatin and release are not specific to collagen-induced platelet activation. These findings strongly support our initial hypothesis that PPAR-β/-γ are involved in nifedipine-mediated down-regulation of CD40L/sCD40L signaling in platelets.

A novel finding of this study is that the suppressive effects of nifedipine on platelet CD40L/sCD40L cascade are PPAR-β/-γ dependent, evidenced by PPAR-β/-γ antagonists diminishing the effects. These results provide a reasonable explanation for the anti-atherosclerotic property of nifedipine. Next, the underlying mechanisms by which PPAR-β/-γ inhibited sCD40L release were investigated. The NO/cyclic GMP pathway is proposed to be responsible for the negative regulation of agonist-induced platelet surface CD40L expression [23]. It has been mentioned previously that PPAR-β/-γ is capable of increasing NO and cyclic GMP formation in platelets by up-regulating PI3K/Akt/eNOS pathway [14]. Our data revealed that nifedipine-mediated elevation of NO and cyclic GMP formation was markedly attenuated by PPAR-β/-γ agonists. Moreover, blocking NO/cyclic GMP formation by using L-NAME and ODQ significantly abolished the decreased CD40L expression and sCD40L release by nifedipine. Altogether, nifedipine inhibits CD40L/sCD40L cascade by means of PPAR-β/-γ -dependent NO/cyclic GMP generation.

Mitogen-activated protein kinases (MAPKs) including p38MAPK, ERK1/2 *(p42/p44 MAPK)* and JNK have been reported to play a critical role in regulating a variety of gene expression and signaling pathways that promote the development of inflammatory and vascular diseases [24]. Notably, MAPKs also exist in human platelets, and stimulate platelet activation by enhancing phospholipase A_2_ (PLA_2_) activity and subsequent thromboxane A_2_ (TXA_2_), a potent activator for the release reaction and aggregation of platelets, formation [25,26]. The phosphorylation of heat shock protein 27 (HSP27) caused by p38MAPK or ERK1/2 is known to induce sCD40L release from collagen-stimulated platelets [32,25] via modulation of actin cytoskeleton [23,27,28]. Parallel to the change of sCD40L release, nifedipine markedly inhibited the phosphorylation of p38MAPK, ERK1/2 and HSP27 but did not affect JNK phosphorylation (data not shown) in collagen-stimulated platelets. In agreement with this evidence that treatment with PPAR-γ agonist or sodium nitroprusside, a nitric oxide donor, reduces the MAPK phosphorylation and the surface of CD40L expression in thrombin-stimulated platelets [23, 29], the above effects of nifedipine were remarkably diminished by GSK0660, GW9662 or L-NAME. Taken together with the results, it is probable that nifedipine-mediated PPAR-β/-γ-dependent NO/cyclic GMP production and down-regulation of MAPks/HSP27 signalling ultimately inhibit the sCD40L release from platelets.

When platelets are activated, arachidonic acid (AA) is liberated from membrane phospholipids through the action of PLA_2_. Then, AA is converted to TXA_2_ by cyclooxygenase (COX) and thromboxane synthase [30]. Recent study has indicated that suppressing TXA_2_ formation by aspirin, a COX inhibitor, significantly inhibited sCD40L release from ristocetin, an activator of GPIIb/IX/V,-stimulated human platelets [31]. Moreover, the elevated plasma levels of sCD40L in atherosclerotic patients were markedly attenuated after aspirin administration [31], strongly supporting the involvement of TXA_2_ in sCD40L release. Based on the previous finding that nifedipine inhibited collagen-induced TXB_2_, a stable metabolite of TXA_2_, generation in platelets [32], the inhibition of TXA_2_ formation may be another mechanism regulating sCD40L release. It is well known that platelet-derived sCD40L release is greatly increased in response to various platelet inducers, suggesting that the sCD40L release from α-granule may be a consequence of platelet activation. In supporting the concept, our previous and supplement data showed that the surface expression of CD62P (S2 Fig), and GPIIb/IIIa that are stored in α-granule are also significantly inhibited by nifedipine in collagen-stimulated platelets [33].

Matrix metalloproteinases (MMPs), a family of zinc and calcium-dependent proteinases, are involved in a variety of biological and pathological effects [34]. During platelet activation, MMP-2 is released from platelets into extracellular microenviroment. Importantly, the action of MMP-2 is considered a critical step for sCD40L release by cleaving the platelet membrane-bound CD40L [10]. The expression and activity of MMP-2 has been reported to be up-regulated by p38MAPK or ERK1/2, while the MMP-2 expression and activity were reduced when p38MAPK or ERK1/2 was inhibited [35]. A recent study showed that administration of pioglitazone is capable of inhibiting MMP-2 activity in peritoneal fibrosis [36], suggesting that the MMP-2 activity is also regulated by PPAR-γ. The results of this study revealed that nifedipine obviously attenuated collagen-induced MMP-2 expression and activity, whereas blocking PPAR-β/-γ activity completely diminished the effects. The ROS, especially hydrogen peroxide, is confirmed to be an important stimulator for MMP-2 activation [37]. Although, the true mechanisms by which ROS enhances MMP-2 activity remain unclear, ROS-induced post-translational modification of MMP-2 via a cysteine switch reaction is proposed to be a possible mechanism [38]. Interestingly, sCD40L also can increase ROS synthesis in activated platelets by activating p38MAPK [39]. Collectively, there is a positive feedback regulating loop existed among ROS, MMP-2 and sCD40L. Our data showed that nifedipine treatment inhibited collagen-induced O_2_^-^ and H_2_O_2_ formation, which was abolished by PPAR-β/-γ antagonists. Therefore, the suppressive effect of nifedipine on MMP-2 expression and activity is ascribed to PPAR-β/-γ -dependent inhibition of p38MAPK and ERK1/2 activation as well as ROS generation, which in turn decreases sCD40L release.

**Fig 6:**
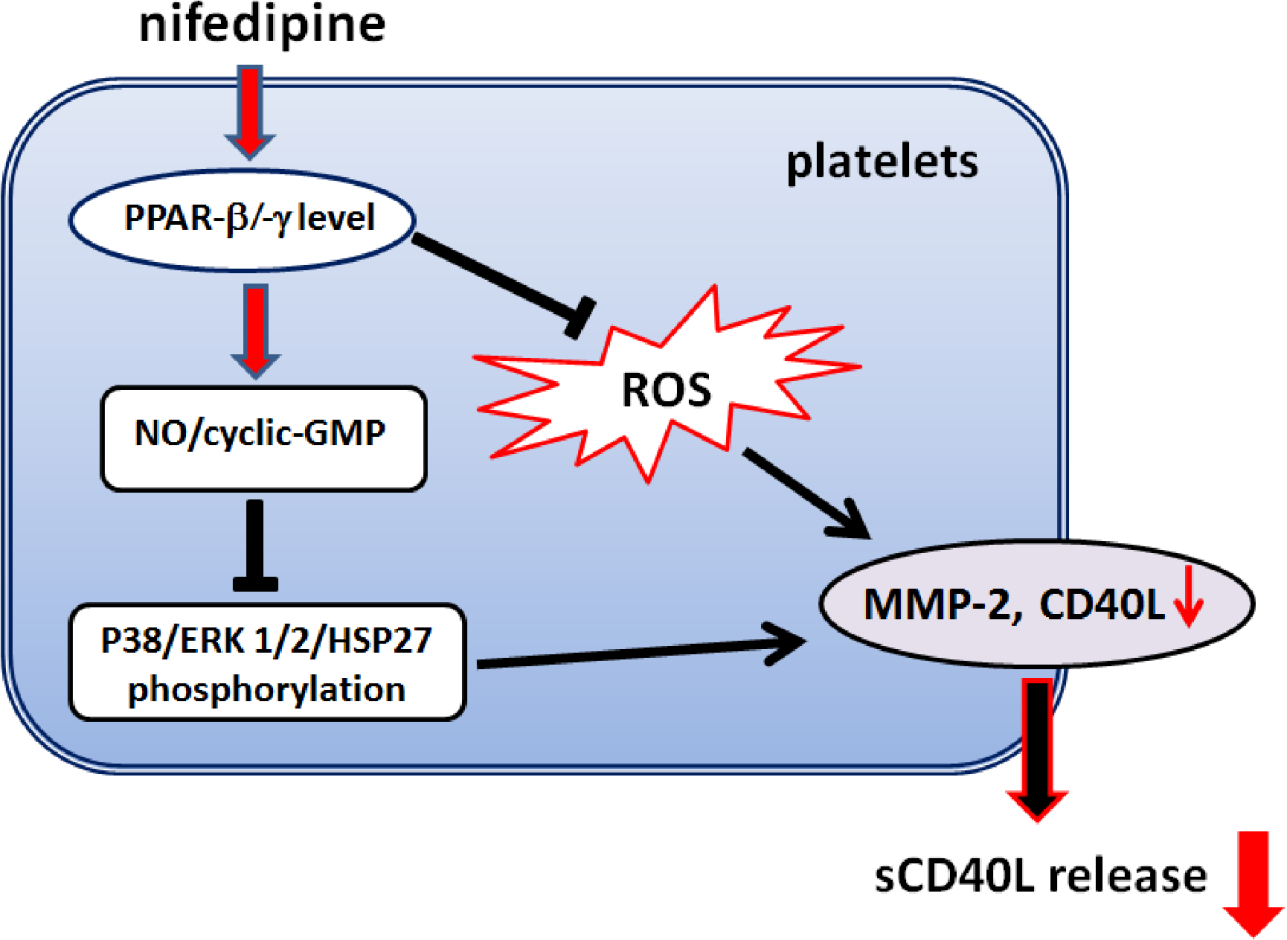
Hypothetical model of the signaling pathways of nifedipine-mediated reduction of sCD40L release from collagen-stimulated platelets. Nifedipine initially activates PPAR-β/-γ followed by increased formation of NO/cyclic GMP and down-regulation of p38MAPK/ERK1/2/HSP27 signaling as well as ROS generation, which then attenuates MMP-2 activity and ultimately inhibits sCD40L release from activated platelets.

In conclusion, as summarized in Fig 6, this study provides the first mechanistic evidence that nifedipine-mediated inhibition of the surface CD40L expression and sCD40L release in activated human platelets is modulated by PPAR-β/-γ. Furthermore, we demonstrated that PPAR-β/-γ -dependent elevation of NO/cyclic GMP formation and downregulation of p38MAPK/ERK1/2/HSP27/MMP-2 signaling pathway and ROS production are key events contributing to suppressing sCD40L release in response to nifedipine. The decreased sCD40L release from platelets is likely to contribute to the protective effect of nifedipine on cardiovascular diseases.

## Supporting Information

S1 Fig Effect of nifedipine on the membrane CD40L expression in thrombin-stimulated platelets. Washed platelets were preincubated with various doses of nifedipine or nifedipine combined with GSK0660 (5 μM) or GW9662 (5 μM) for 3 min, followed by addition of thrombin (0.8 U/ml) for 6 min. The plot representative of three or four independent experiments. A: resting platelets, B: thrombin-stimulated alone platelets, C: thrombin+Nif (1 μM), D: thrombin+Nif (5 μ M), E: thrombin+Nif (5 μM)+GSK0660, F: thrombin+Nif (5 μM)+GW9662.
(TIF)

S2 Fig Effect of nifedipine on the membrane CD62P expression in collagen-stimulated platelets. Washed platelets were preincubated with various concentrations of nifedipine for 3 min, followed by addition of collagen (10 μg/ml) for 6 min. The platelet surface CD62P was determined. The plot is representative of three or four independent experiments. A: resting platelets, B: collagen-stimulated alone platelets, C: collagen+Nif (1 μM), D: collagen+Nif (5 μM).
(TIF)
