# Supplementary figures and images for: Mechanisms of Nifedipine-Downregulated CD40L/sCD40L Signaling in Collagen Stimulated Human Platelets
Source: PLoS One. 2015 May 13;10(5):e0127054. doi: 10.1371/journal.pone.0127054 (PMC4430314; doi:10.1371/journal.pone.0127054)

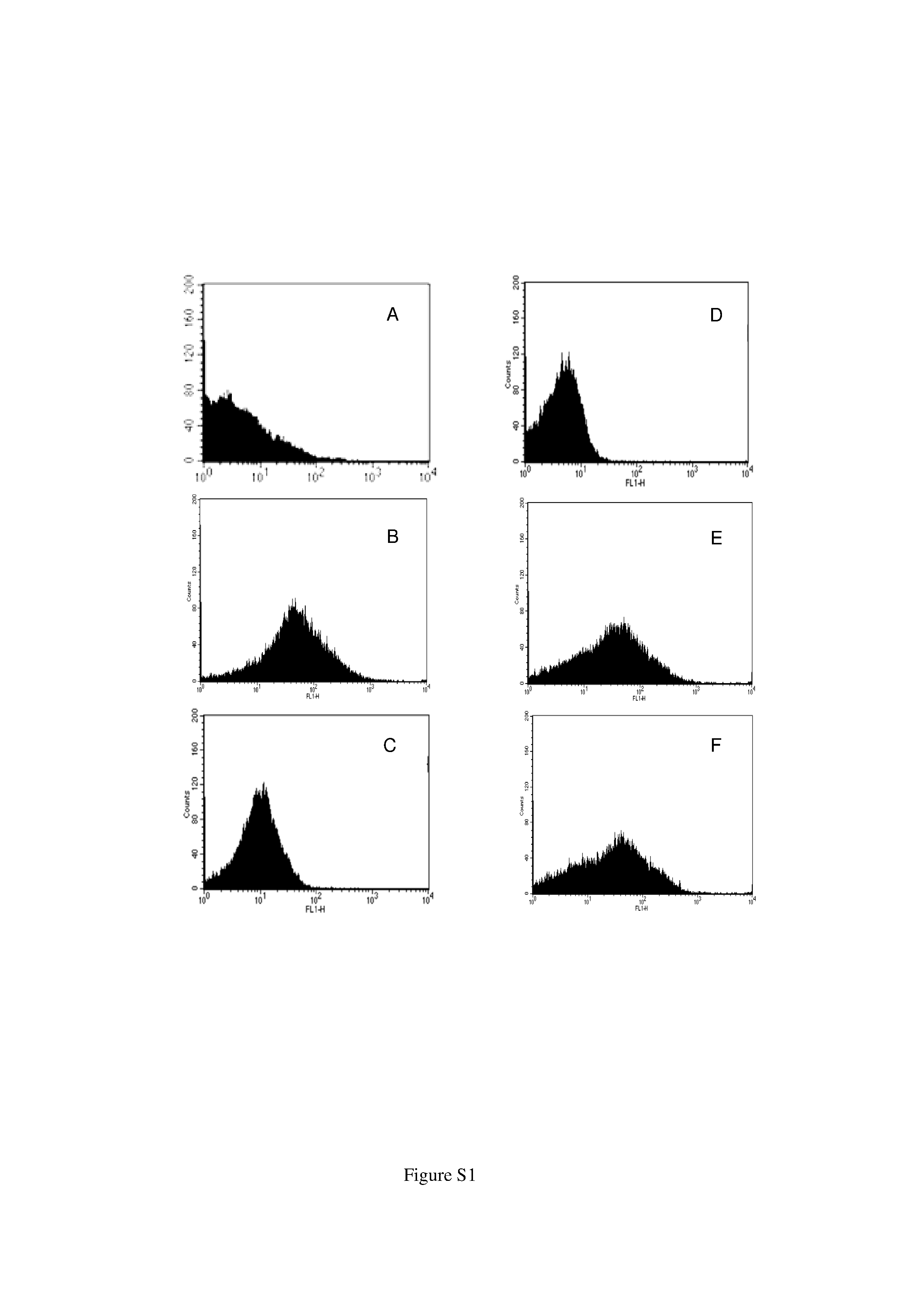

Supplement: S1 Fig — Washed platelets were preincubated with various doses of nifedipine or nifedipine combined with GSK0660 (5 μM) or GW9662 (5 μM) for 3 min, followed by addition of thrombin (0.8 U/ml) for 6 min. The plot representative of three or four independent experiments. A: resting platelets, B: thrombin-stimulated alone platelets, C: thrombin+Nif (1 μM), D: thrombin+Nif (5 μ M), E: thrombin+Nif (5 μM)+GSK0660, F: thrombin+Nif (5 μM)+GW9662. (TIF) [file pone.0127054.s001.tif]

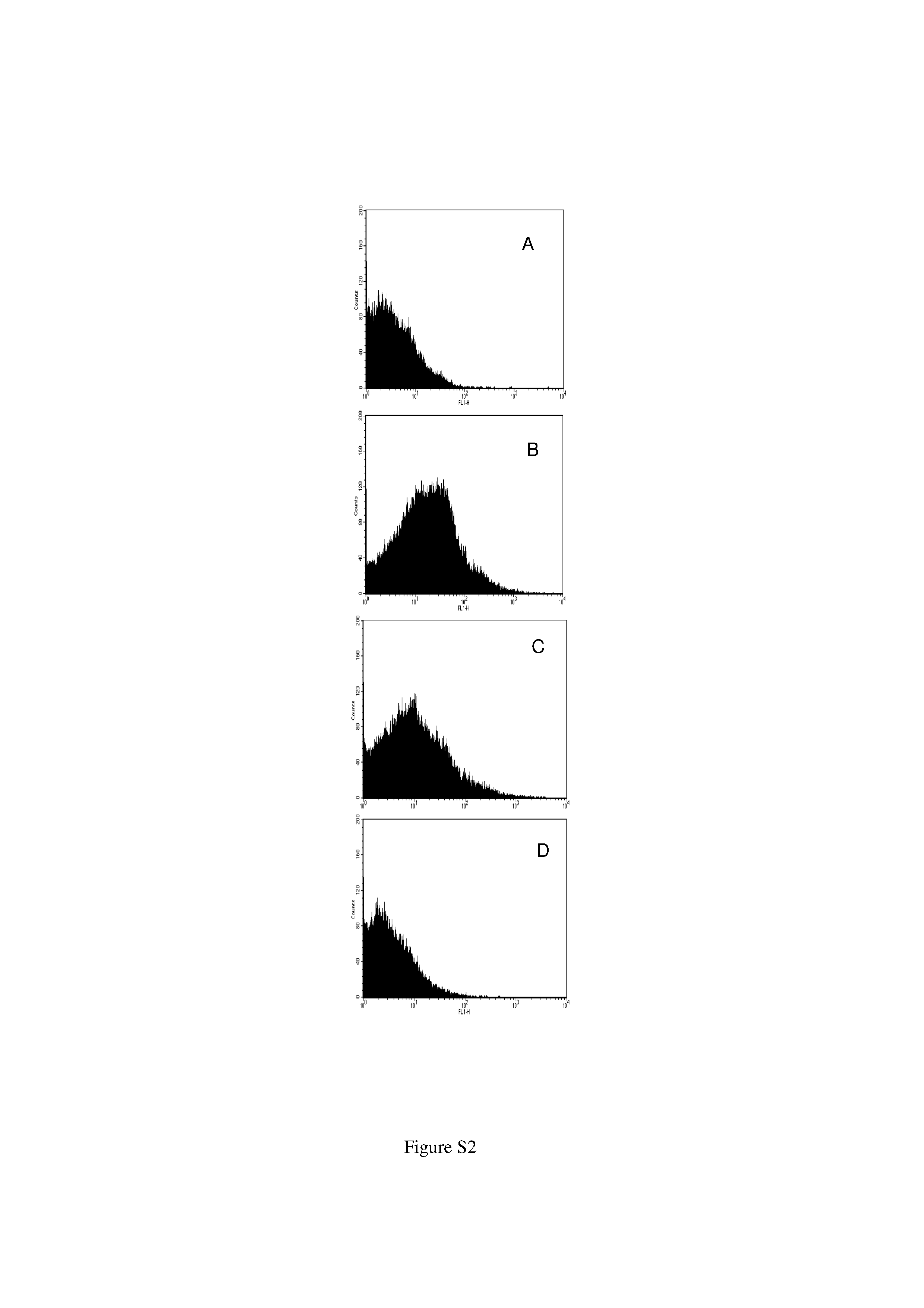

Supplement: S2 Fig — Washed platelets were preincubated with various concentrations of nifedipine for 3 min, followed by addition of collagen (10 μg/ml) for 6 min. The platelet surface CD62P was determined. The plot is representative of three or four independent experiments. A: resting platelets, B: collagen-stimulated alone platelets, C: collagen+Nif (1 μM), D: collagen+Nif (5 μM). (TIF) [file pone.0127054.s002.tif]
